# Twin Pregnancy Complicated by Gestational Diabetes Mellitus: Maternal and Neonatal Outcomes

**DOI:** 10.1210/jendso/bvae075

**Published:** 2024-04-16

**Authors:** Devika Das, Hannah E Christie, Moustafa Hegazi, Marina Takawy, Karina A Pone, Adrian Vella, Aoife M Egan

**Affiliations:** Department of Internal Medicine, Mayo Clinic, Rochester, MN 55905, USA; Department of Endocrinology, Diabetes, Metabolism and Nutrition, Mayo Clinic, Rochester, MN 55905, USA; Department of Endocrinology, Diabetes, Metabolism and Nutrition, Mayo Clinic, Rochester, MN 55905, USA; Department of Endocrinology, Diabetes, Metabolism and Nutrition, Mayo Clinic, Rochester, MN 55905, USA; Division of Maternal and Fetal Medicine, Mayo Clinic, Rochester, MN 55905, USA; Department of Endocrinology, Diabetes, Metabolism and Nutrition, Mayo Clinic, Rochester, MN 55905, USA; Department of Endocrinology, Diabetes, Metabolism and Nutrition, Mayo Clinic, Rochester, MN 55905, USA

**Keywords:** pregnancy, twin, diabetes, hyperglycemia, gestational diabetes

## Abstract

**Context:**

The risk of gestational diabetes mellitus (GDM) in twin pregnancies is more than double that of singleton pregnancies. Although twin pregnancies present unique challenges for fetal growth and prenatal management, the approach to GDM diagnosis and treatment is the same regardless of plurality. Data on pregnancy outcomes for individuals with GDM and a twin pregnancy are limited and conflicting.

**Objective:**

To describe the maternal characteristics associated with GDM in twin pregnancies and to assess the associated pregnancy outcomes compared to twin pregnancies unaffected by GDM.

**Methods:**

A retrospective cohort study was conducted at Mayo Clinic, Rochester, Minnesota, USA, and included predominantly Causasian women aged 18 to 45 years who received prenatal care for a twin pregnancy from 2017-2022. Maternal characteristics and a broad spectrum of pregnancy outcomes were evaluated. Universal GDM screening involved a 50 g oral glucose challenge test +/− a 100 g oral glucose tolerance test.

**Results:**

GDM was diagnosed in 23% pregnancies (n = 104/452). Compared to those without, women with GDM had known risk factors including a higher prepregnancy body mass index (31.1vs 26.3 kg/m^2^; *P* < .01) and a prior history of GDM (21.7 vs 5.9%; *P* < .01). There were no differences in maternal pregnancy complications or neonatal outcomes between groups. Attendance at postpartum glucose testing among women with GDM was poor at 27.9% (29/104).

**Conclusion:**

These data suggest that women with twin pregnancies share a similar GDM risk profile to those with singleton pregnancies and provide reassuring evidence that current management for GDM twin pregnancies produces similar outcomes to twin pregnancies without GDM.

The incidence of both gestational diabetes mellitus (GDM) and twin pregnancies has increased over the past 2 decades, in part due to common risk factors such as increased rates of obesity, advanced maternal age, and use of advanced reproductive technologies [1]. GDM is reported to be increased in twin pregnancies compared to singleton pregnancies [2], possibly due to the aforementioned maternal demographics and increased placental size [3, 4].

GDM and twin pregnancies are independently associated with several maternal and neonatal complications [3, 5, 6]. However, twin pregnancies frequently result in both iatrogenic and spontaneous preterm deliveries and lower birthweight infants, which may in turn reduce the incidence of known GDM risks such as accelerated fetal growth and shoulder dystocia [3]. On the contrary, it is also possible that GDM in a twin pregnancy could exacerbate adverse outcomes, including increased rates of induction, increased perinatal mortality rate, and preeclampsia [7, 8].

In the United States, a 2-step screening approach is commonly used to diagnose GDM [9, 10], whereas a 1-step approach is favored in other locations [11]. Notwithstanding the choice of approach, clinicians generally apply their choice of screening criteria and treatment targets irrespective of fetal count. Data on clinical outcomes for twin pregnancies with maternal GDM are limited and often conflicting, impacting our understanding of optimal treatment recommendations [1, 6-8, 12-16].

The aim of this study is to describe the maternal demographics and clinical outcomes for twin pregnancies affected by GDM and compare this to twin pregnancies unaffected by GDM using 5-year data from Mayo Clinic, an academic hospital with approximately 2400 deliveries per year.

## Material and Methods

We performed a retrospective cohort study after approval from the Mayo Clinic Institutional Review Board. We conducted a search of the Mayo Clinic electronic medical record system using Mayo Data Explorer, a Mayo Clinic-developed data exploration and retrieval tool that facilitates searching of data from multiple clinical and hospital source systems within Mayo Clinic. A major source of data is Mayo Clinic's Unified Data Platform data warehouse environment. The Unified Data Platform was created from multiple source systems to provide researchers access to Mayo Clinic–wide clinical data in a single centralized database. These source systems include electronic and scanned medical records including patient demographics, diagnoses, hospital and outpatient clinical notes, and laboratory data. We conducted an initial broad search using the following criteria: all diagnostic codes containing “twin” AND all diagnostic codes containing “pregnancy” AND gender equal to “female” “unknown” or “other” AND “age ≥ 18 years” AND “age ≤ 45 years.” Individuals without research authorization were excluded. By manual review of this list, we isolated individuals between the ages of 18 and 45 years who had twin pregnancies with delivery dates from January 1, 2017, to June 1, 2022, with and without GDM. The study start date reflects the timepoint when source system labs and diagnostic codes became reliably retrievable using Mayo Data Explorer. We identified 452 twin pregnancies, of which 104 (23%) were complicated by a diagnosis of GDM. In addition to crosschecking electronically retrieved data, additional demographic information and clinical variables were manually extracted directly from the electronic medical record for each individual by 3 physicians (D.D., M.H., M.T.) following a standardized protocol with a fourth physician (A.M.E.) cross-checking the abstracted data for accuracy.

## Clinical Practice

Universal screening for GDM occurs at our institution with a 1-hour 50 g glucose challenge test between 24 and 28 weeks of gestation. If the result is ≥ 190 mg/dL, the patient is assumed to have GDM without pursuing a confirmatory 3-hour test. If the result is ≥ 140 to 190 mg/dL, a 3-hour 100 g glucose challenge test is recommended. GDM is diagnosed based on the ≥ 2 thresholds being equal to or exceeding the Carpenter and Coustan criteria for GDM [9]. Following diagnosis, individuals with GDM meet with a diabetes nurse specialist and registered dietician who provide education and medical nutritional therapy. They are advised to test their glucose fasting in the morning and either 1 or 2 hours after each meal with a goal glucose of <95 mg/dL fasting, < 140 mg/dL 1 hour post prandially, or <120 mg/dL 2 hours post prandially. On average, if >20% values are above goal, pharmacological therapy is initiated, with insulin being the preferred choice.

## Demographic Measurements

The following maternal characteristics were collected: maternal age, prepregnancy body mass index (BMI), parity, and chorionicity. BMI was recorded as the BMI at the time of the first obstetric visit for the corresponding pregnancy or the prepregnancy BMI within 3 months of pregnancy confirmation, if available. Individuals were classified as underweight if the BMI was < 18.5 kg/m^2^, normal if the BMI was between 18.5 and 24.9 kg/m^2^, overweight if the BMI was between 25.0 and 29.9 kg/m^2^, and obese if the BMI was greater than or equal to 30.0 kg/m^2^ [17]. Fertility treatment included ovarian stimation, intrauterine insemination, and/or intravenous fertilization.

## Outcome Variables

Outcomes were selected based on review of prior studies in this area and a relevant core outcome set [18]. The following maternal complications were documented: chronic hypertension, gestational hypertension/preeclampsia, and cesarean delivery. Chronic hypertension was defined as hypertension diagnosed or present before pregnancy (systolic blood pressure 130-139 mmHg or diastolic 80-89 mmHg or systolic blood pressure systolic blood pressure ≥ 140 mmHg and/or diastolic blood pressure ≥ 90 mmHg) or on at least 2 occasions before 20 weeks of gestation (systolic blood pressure ≥ 140 mmHg and/or diastolic blood pressure ≥ 90 mmHg) [19]. Gestational hypertension was defined as the new onset of hypertension (systolic blood pressure ≥ 140 mmHg and/or diastolic blood pressure ≥ 90 mmHg) at 20 weeks or more of gestation in the absence of proteinuria or new signs of end-organ dysfunction [20]. Preeclampsia was defined as the new onset of hypertension and proteinuria or the new onset of hypertension and significant end-organ dysfunction with or without proteinuria after 20 weeks of gestation [20].

The following neonatal outcomes were collected: gestational age, sex, birthweight, APGAR score at 1 minute, admission to the neonatal intensive care unit (NICU), presence of a congenital anomaly, neonatal hypoglycemia, and breastfeeding during the postpartum hospitalization. Fetal demise was defined as delivery of a fetus at 20 weeks or more of gestation with no signs of life, and neonatal death was defined as the death of a liveborn infant within the first 28 days of life. Miscarriages (pregnancy loss before 20 weeks gestation) were excluded from the data collection as the diagnosis of GDM occurs after the loss of a pregnancy. Neonates were defined as small for gestational age (SGA) or large for gestational age (LGA) when their birthweight was below the 10th or above the 90th percentile according to the 2017 US reference for singleton birthweight percentiles using obstetric estimates of gestation and adjusted for gestational age of birth and sex [21]. Congenital anomalies were documented if meeting the criteria of the Centers for Disease Control and Prevention selected external and internal major congenital anomalies as these account for most of the deaths, morbidity, and disability related to congenital anomalies [22]. Neonatal hypoglycemia was defined as a neonatal glucose less than or equal to 45 mg/dL (2.5 mmol/L) and administration of any treatment for hypoglycemia.

Follow-up maternal data of the twin GDM pregnancies were collected including completion and results of a 6- to 12-week postpartum glucose tolerance test and diagnosis of diabetes or prediabetes on the postpartum glucose tolerance test. Prediabetes was diagnosed on the postpartum glucose test if the fasting plasma glucose was 100 to 125 mg/dL or the 2-hour plasma glucose was 140 to 199 mg/dL. Type 2 diabetes mellitus was diagnosed on the postpartum glucose test if the fasting plasma glucose was ≥ 126 mg/dL or if the 2-hour plasma glucose was ≥ 200 mg/dL. If a subsequent pregnancy was recorded (last assessment in January 2023), the presence or absence of GDM was noted. Incident diabetes or prediabetes diagnosed during this postpartum timeframe was also recorded.

Data were analyzed using BlueSky Statistics (commercial server edition, version 10.3.1). Comparisons between twin pregnancies with and without GDM were evaluated using the Chi-square or Fisher’s exact test, as appropriate, for categorical variables. Comparisons between the GDM twin pregnancies and non-GDM twin pregnancies were evaluated using the Wilcoxon Mann–Whitney test for continuous variables that were not normally distributed based on histograms and Shapiro–Wilk testing and the 2-sample *t*-test for continuous variables that were normally distributed.

## Results

### Maternal Characteristics

A total of 104 pregnancies with and 348 pregnancies without GDM were analyzed. Table 1 describes maternal characteristics for all pregnancies, according to GDM status. There was no difference in maternal age between groups. Race distribution was also similar between the groups, with the majority being Caucasian, similar to the background population in our region. The median prepregnancy BMI was significantly higher in the GDM group at 31.1 kg/m^2^ compared to 26.3 kg/m^2^ in the non-GDM group (*P* < .0001) with an increased percentage of individuals with an obese BMI (56.8% in GDM vs 28.0% in non-GDM, *P* < .0001).

**Table 1.** Maternal characteristics

	GDM Twin pregnancies n = 104	non-GDM Twin pregnancies n = 348	*P* (GDM vs non-GDM twin pregnancies)
Maternal age (years)	31.0 (28.0-34.0)	30.0 (27.0-33.0)	.06
Maternal age ≥ 35 years	20 (19.2)	70 (20.1)	.84
Race			
Caucasian	93 (89.4)	297 (85.3)	.71
Black	5 (4.8)	24 (6.9)	
Asian	3 (2.9)	13 (3.7)	
Other	1 (1.0)	10 (2.9)	
Chose not to disclose	2 (1.9)	4 (1.1)	
Prepregnancy BMI (kg/m^2^)*^a^*	31.1 (25.3-36.9)	26.3 (23.2-31.2)	**<**.**0001**
Underweight	0 (0)	1 (0.4)	**<**.**0001**
Normal weight	21 (22.1)	97 (39.4)	
Overweight	20 (21.1)	79 (32.1)	
Obese	54 (56.8)	69 (28.0)	
Parity	1 (0-2)	1 (0-2)	.48
Parity 0	44 (42.3)	126 (36.2)	.42
Parity 1	28 (26.9)	115 (33.0)	
Parity > 1	32 (30.8)	107 (30.7)	
Chorionicity			
Monochorionic monoamniotic	1 (1.0)	1 (0.3)	.39
Monochorionic diamniotic	22 (21.2)	79 (22.7)	
Dichorionic diamniotic	81 (77.9)	262 (75.3)	
Unknown	0 (0)	6 (1.7)	
Previous GDM	13 (12.5)	13 (3.7)	.**0008**
Fertility treatment	30 (28.8)	79 (22.7)	.20
Smoking during pregnancy	5 (4.8)	30 (8.6)	.29
50g glucose challenge test completed	91 (87.5)	276 (79.3)	.06
Glucose challenge result			
(mmol/L)	9.1 (8.4-9.9)	6.8 (5.8-7.8)	**<**.**0001**
(mg/dL)	165.0 (151.0-179.0)	122.0 (103.8-140.8)	
100g 3-hour glucose tolerance test completed	74 (71.2)	63 (18.0)	**<**.**001**
Fasting glucose			
(mmol/L)	4.8 (4.6-5.2)	4.6 (4.4-4.8)	.**0008**
(mg/dL)	87.0 (82.0-93.0)	82.0 (79.0-87.0)	
1-hour glucose			
(mmol/L)	10.6 (10.0-11.3)	8.7 (7.8-9.5)	
(mg/dL)	190.5 (180.3-203.3)	157.0 (140.0-171.0)	**<**.**0001**
2-hour glucose			
(mmol/L)	9.4 (8.9-10.3)	7.2 (6.8-8.0)	
(mg/dL)	170.0 (161.0-185.0)	130.0 (123.0-143.5)	**<**.**0001**
3-hour glucose			
(mmol/L)	7.4 (6.1-8.2)	5.6 (4.8-6.8)	
(mg/dL)	133.0 (109.0-147.0)	100.0 (86.0-121.5)	**<**.**0001**

Data presented as median (quartile 1-quartile 3) or n (%). Statistically significant findings are shown in bold.

Abbreviations: BMI, body mass index; GDM, gestational diabetes mellitus.

^*a*^Data available on n = 95 with GDM and n = 246 without GDM.

The median parity in both groups was 1 with a range of 0 to 6 in the GDM group and 0 to 7 in the non-GDM group (*P* = .48). The majority of pregnancies in both groups were dichorionic diamniotic (77.9% in GDM and 75.3% in non-GDM, *P* = .39). Individuals with GDM were more likely to have a history of previous GDM (12.5% with GDM vs 3.7% without, *P* < .001). A high proportion of mothers underwent fertility treatments in both groups (28.8% in GDM vs 22.7% in non-GDM, *P* = .20), and there was no significance in the proportion who reported smoking during pregnancy (4.8% in GDM vs 8.6% in non-GDM, *P* = .29).

A 50 g glucose challenge test was documented in 87.5% of GDM pregnancies and 79.3% of non-GDM pregnancies (*P* = .06). The median glucose challenge result was 165 mg/dL in the GDM group and 122 mg/dL in the non-GDM group (*P* < .0001). For those who completed a subsequent 3-hour oral glucose tolerance test, the median values for each timepoint were higher in the group with GDM. Pharmacological therapy for GDM was required in 35.6% of GDM pregnancies.

### Maternal Outcomes

Table 2 outlines key maternal complications during pregnancy. Chronic hypertension was present in 4.8% of GDM pregnancies and 2.9% of non-GDM pregnancies (*P* = .35). There were similar but high rates of gestational hypertension/preeclampsia and cesarean delivery in both groups. A majority of mothers in both the GDM and non-GDM groups breastfed during their postpartum hospitalization (94.2% in GDM vs 89.9% in non-GDM, *P* = .41).

**Table 2.** Maternal outcomes

	GDM Twin pregnancies n = 104	non-GDM Twin pregnancies n = 348	*P* (GDM vs non-GDM twin pregnancies
Chronic hypertension	5 (4.8)	10 (2.9)	.35
Gestational hypertension/preeclampsia	34 (32.7)	101 (29.0)	.47
Cesarean delivery	79 (76.0)	238 (68.4)	.17
Breastfeeding	98 (94.2)	313 (89.9)	.41

Data presented as n (%).

Abbreviations: GDM, gestational diabetes mellitus.

### Fetal and Neonatal Outcomes

Table 3 summarizes the fetal and neonatal outcomes in those with and without GDM. Fetal demise (0.5% in GDM vs 1.3% in non-GDM, *P* = .47) and neonatal death (1.0% in GDM vs 1.9% in non-GDM, *P* = .54) occurred at similar rates in both groups. After exclusion of fetal demise, 207 neonates in the GDM group and 687 neonates in the non-GDM group were further evaluated. The median gestational age at delivery in both groups was 36 weeks (*P* = .44). The majority of deliveries took place prematurely in both groups, either before 37 weeks (65.2% in GDM vs 60.3% in non-GDM, *P* = .20) or before 34 weeks (16.4% in GDM vs 18.9% in non-GDM, *P* = .42). Approximately half of the neonates in both groups were female (46.9% in GDM vs 51.8% in non-GDM, *P* = .21). There was no difference in birthweight between groups. A similar percentage of neonates weighed < 2.5 kg in both groups (51.2% in GDM vs 49.65 in non-GDM, *P* = .46). In addition, a similar percentage of neonates were found to be LGA (0.5% in GDM vs 3.2% in non-GDM) and SGA (15.9% in GDM vs 16.7% in non-GDM) (*P* = .08). The median 1-minute APGAR score in both groups was 8 (*P* = .50).

**Table 3.** Fetal and neonatal outcomes

	GDM Twin pregnancies n = 208	non-GDM Twin pregnancies n = 696	*P* (GDM vs non-GDM twin pregnancies)
Livebirth	207 (99.5)	687 (98.7)	.47
Fetal demise	1 (0.5)	9 (1.3)	
Neonatal death	2 (1.0)	13 (1.9)	.54
	**GDM livebirths** **n = 207**	**Non-GDM livebirths** **n = 687**	
Gestational week at delivery	36 (34-37)	36 (34-37)	.44
Delivery < 37 weeks	135 (65.2)	414 (60.3)	.20
Delivery < 34 weeks	34 (16.4)	130 (18.9)	.42
Female sex	97 (46.9)	356 (51.8)	.21
Birthweight (kg)	2.4 (2.1-2.8)	2.5 (2.1-2.8)	.69
Birthweight >4.0kg	0 (0)	0 (0)	
Birthweight <2.5kg	106 (51.2)	341 (49.6)	.46
LGA	1 (0.5)	22 (3.2)	.08
SGA	33 (15.9)	115 (16.7)	
AGA	167 (80.7)	548 (79.8)	
APGAR at 1 minute	8 (6-8)	8 (8-9)	.50
	**n = 166*^a^***	**n = 658*^a^***	
Admission to NICU	72 (43.4)	243 (36.9)	.13
Congenital anomaly	16 (9.6)	38 (5.8)	.08
Neonatal hypoglycemia	73 (44.0)	291 (44.2)	.52

Data presented as median (quartile 1-quartile 3) or n (%).

Abbreviations: AGA, appropriate for gestational age; GDM, gestational diabetes mellitus; LGA, large for gestational age; NICU, neonatal intensive care unit; SGA, small for gestational age.

^*a*^Absent research authorization for neonates resulted in a reduced denominator.

Admission to the NICU occurred in 43.4% of neonates in the GDM group and 36.9% in the non-GDM group (*P* = .13). The incidence of congenital anomaly was 9.6% in the GDM group and 5.8% in the non-GDM group (*P* = .08). The most common congenital anomalies were cardiac, particularly atrial septal defects. The percentage of neonatal hypoglycemia was 44.0% in the GDM group and 44.2% in the non-GDM group (*P* = .52).

### Postpartum Follow-up of Individuals With GDM

Table 4 outlines postpartum follow-up of those with a twin pregnancy affected by GDM. The postpartum glucose tolerance test was completed in a minority (27.9%) of those with GDM, with a mean fasting glucose of 87.9 mg/dL and mean 2-hour glucose of 101.4 mg/dL. Diabetes or prediabetes was diagnosed in 10.3% of individuals. Subsequent pregnancies were recorded in 17.3% of individuals with a twin GDM pregnancy, and of those 44.4% were diagnosed with GDM in a subsequent pregnancy. Most (90.4%) remained a resident of the area, and incident diabetes or prediabetes was diagnosed in 11.5%.

**Table 4.** Postpartum follow up of individuals with GDM

	GDM Twin pregnancies n = 104
Postpartum glucose tolerance test completed	29 (27.9)
Fasting glucose	
(mmol/L)	4.8 (4.7-5.1)
(mg/dL)	87.0 (84.0-92.0)
2-hour glucose	
(mmol/L)	5.6 (4.7-6.2)
(mg/dL)	100.0 (84.8-111.5)
Diabetes or prediabetes diagnosed on postpartum glucose tolerance test*^a^*	3 (10.3)
Subsequent pregnancy recorded	18 (17.3)
GDM in subsequent pregnancy*^b^*	8 (44.4)
Still resident	94 (90.4)
Incident diabetes or prediabetes diagnosed on subsequent follow up	12 (11.5)

Data presented as median (quartile 1-quartile 3) or n (%).

Abbreviations: GDM, gestational diabetes mellitus.

^*a*^Denominator is 29, reflecting those that completed an oral glucose tolerance test.

^*b*^Denominator is 18, reflecting those that had a subsequent pregnancy recorded.

## Discussion

This study provides data on key maternal and neonatal characteristics and outcomes for 104 twin pregnancies with GDM and 348 twin pregnancies without GDM over a 5-year period at a large academic institution. Although at 23%, the prevalence of GDM in the twin pregnancy population is more than double that of singleton populations [23], maternal characteristics are similar. These include increased BMI, a prior history of GDM, and a similar risk of GDM recurrence [18, 24]. In the context of a standard approach to GDM treatment, individuals both with and without GDM in a twin pregnancy experienced similar risk of maternal complications, including cesarean delivery and preeclampsia/gestational hypertension. In addition, neonatal outcomes were similar between groups, including birthweight, rates of NICU admissions, congenital anomalies, and the presence of hypoglycemia.

Prior studies have examined the association between GDM and adverse outcomes in twin pregnancies. Data on outcomes are conflicting and likely reflect differences in screening and treatment of GDM, along with variable study design and study size limitations (often case series) [1, 6-8, 12-16]. This is highlighted in a recent systematic review and meta-analysis of GDM in twin pregnancy that noted high between-study heterogeneity with significant methodological variation, with the authors calling for caution when interpreting the results [14]. Nonetheless, our results overall support a key takeaway suggesting that GDM in twin pregnancies may have a milder effect on some adverse perinatal outcomes than GDM in singleton pregnancies [14]. However, the authors also reported an increased risk of hypertensive disorders of pregnancy, Caesarean delivery, LGA births, preterm births, and admission to the NICU in twin pregnancies with GDM [14], which was not seen in our study. One of the larger studies in this meta-analysis found that GDM in twins was not associated with hypertensive complications but was associated with increased risk of cesarean delivery, preterm birth, and LGA birthweight [12]. However, this study was confounded by the use of 2 different GDM diagnostic criteria within the study period, both of which differed from the Carpenter and Coustan approach used in this current study [9]. In addition, although a population-based study, approximately 50% were excluded due to missing data on key variables.

Twin pregnancies have been associated with increased risk of fetal growth restriction, posing the possibility that GDM may have a positive impact in decreasing this risk [5, 12, 25-27]. In addition, it has been considered that improved glycemic control in twin pregnancies with GDM does not improve outcomes and is instead associated with a higher risk of SGA [13]. Our data do not reflect this, as there was no difference in the percentage of SGA neonates between the groups, and the majority of neonates were found to have normal weights for their gestational age. This provides some reassurance that we are not overtreating those diagnosed with GDM in twin pregnancies. Nevertheless, future work should address optimal glycemic goals for this population, particularly considering the baseline risk of low birthweight and premature neonates in twin pregnancies overall [28, 29].

In our study population, pregravid obesity emerges as a significant risk factor for GDM during twin pregnancy, in agreement with prior studies in this population [3, 6]. Unfortunately, gestational weight gain could not be accurately documented in this study. While excessive gestational weight gain is well recognized as a GDM risk factor in singleton pregnancies [30, 31], data on the effect of gestational weight gain and clinical guidance for twin pregnancies are limited [32]. One prior study evaluated the extent to which midpregnancy weight gain predisposes an individual to developing GDM in twin pregnancies but did not find a significant effect [33]. Furthermore, while is it generally agreed that individuals pregnant with twins should increase their energy intake, more precise recommendations considering factors such as GDM, preprengancy BMI, and gestational weight gain are lacking [34, 35]. While additional data on gestational weight gain in twin pregnancy is awaited, we recommend a clinical focus on prepregnancy planning with the aim of attaining a healthy BMI before conception.

A notable finding from our study is that only 27.9% of individuals diagnosed with GDM during their twin pregnancy completed a postpartum glucose tolerance test, and over a third were diagnosed with prediabetes or type 2 diabetes based on that test. Furthermore, almost half (44.4%) developed GDM when a subsequent pregnancy was recorded, similar to our singleton population [18]. While poor attendance at postpartum glucose screening is well described [36], we need to renew efforts to overcome barriers to attendance and facilitate postpartum engagement with this high-risk group.

Although our study is limited by its retrospective nature, our cohort contains detailed patient-level data obtained through careful abstraction from an electronic medical record. In this respect our data set is very comprehensive, with less than 5% missing data across the majority of end points collected. Maternal prepregnancy BMI is a notable exception as highlighted in Table 1. Our obstetric practice serves the local population, which is made up of predominantly Caucasian women, and this is reflected in our study demographics. While this could limit the generalizability of our findings, we anticipate that the majority of our findings will be applicable to all individuals with GDM in twin pregnancy. We did not compare clinical outcomes for our twin pregnancy population to our singleton population with or without GDM, but this could be a focus of future work. Finally, our sample size was dictated by our study period, which was defined by consistent availability of electronic medical data. As a result, our power to detect significant between-group differences in rare outcomes such as neonatal death was limited.

In conclusion, our study shows that while GDM affects almost 1 in 4 twin pregnancies, our standard approach to diagnosis and treatment results in clinical outcomes similar to twin pregnancies without GDM. Women affected by GDM during a twin pregnancy have similar clinical risk factors to those diagnosed during a singleton pregnancy and are at equally high risk of future GDM and postpartum glucose intolerance.

## Data Availability

Some or all data sets generated during and/or analyzed during the present study are not publicly available but are available from the corresponding author on reasonable request.

## Abbreviations

BMI: body mass index
GDM: gestational diabetes mellitus
LGA: large for gestational age
NICU: neonatal intensive care unit
SGA: small for gestational age
